# Genomic prediction from observed and imputed high-density ovine genotypes

**DOI:** 10.1186/s12711-017-0315-4

**Published:** 2017-04-20

**Authors:** Nasir Moghaddar, Andrew A. Swan, Julius H. J. van der Werf

**Affiliations:** 1 Cooperative Research Centre for Sheep Industry Innovation, Armidale, NSW 2351 Australia; 2 School of Environmental and Rural Science, University of New England, Armidale, NSW 2351 Australia; 3 Animal Genetics and Breeding Unit (AGBU), University of New England, Armidale, NSW 2351 Australia

## Abstract

**Background:**

Genomic prediction using high-density (HD) marker genotypes is expected to lead to higher prediction accuracy, particularly for more heterogeneous multi-breed and crossbred populations such as those in sheep and beef cattle, due to providing stronger linkage disequilibrium between single nucleotide polymorphisms and quantitative trait loci controlling a trait. The objective of this study was to evaluate a possible improvement in genomic prediction accuracy of production traits in Australian sheep breeds based on HD genotypes (600k, both observed and imputed) compared to prediction based on 50k marker genotypes. In particular, we compared improvement in prediction accuracy of animals that are more distantly related to the reference population and across sheep breeds.

**Methods:**

Genomic best linear unbiased prediction (GBLUP) and a Bayesian approach (BayesR) were used as prediction methods using whole or subsets of a large multi-breed/crossbred sheep reference set. Empirical prediction accuracy was evaluated for purebred Merino, Border Leicester, Poll Dorset and White Suffolk sire breeds according to the Pearson correlation coefficient between genomic estimated breeding values and breeding values estimated based on a progeny test in a separate dataset.

**Results:**

Results showed a small absolute improvement (0.0 to 8.0% and on average 2.2% across all traits) in prediction accuracy of purebred animals from HD genotypes when prediction was based on the whole dataset. Greater improvement in prediction accuracy (1.0 to 12.0% and on average 5.2%) was observed for animals that were genetically lowly related to the reference set while it ranged from 0.0 to 5.0% for across-breed prediction. On average, no significant advantage was observed with BayesR compared to GBLUP.

## Background

The development of high-throughput genotyping based on single nucleotide polymorphisms (SNPs) in livestock species has made the implementation of genomic evaluation more practical. In genomic prediction, the breeding values of selection candidates are evaluated according to their genotypes and a prediction equation derived from a reference population with both phenotypes and genotypes [1]. The accuracy of genomic prediction relies on several factors including linkage disequilibrium (LD) between genome-wide SNPs and quantitative trait loci (QTL) that are responsible for the phenotypic variation of traits of interest [1]. High-density (HD) SNP genotypes can result in stronger LD between SNPs and QTL which can improve the accuracy of genomic prediction in livestock, e.g. [2–5].

Results of simulation studies in livestock show various degrees of improvement in genomic prediction when using HD genotypes compared to genotypes from moderate-density SNP panels such as 50k. For example, based on simulation studies, Meuwissen and Goddard [6] reported a large gain (>40%) in prediction accuracy from HD genotypes, while VanRaden et al. [7] and Harris and Johnson [8] found zero to only small gains in prediction accuracy. Such differences can be attributed to the assumption made about the distribution of QTL effects in the simulated models. Meuwissen and Goddard [6] and Clark et al. [9] showed that both the number and distribution of QTL effects that control a polygenic trait have a significant impact on the advantage of using HD genotypes in genomic prediction, with only small benefits for the “infinitesimal’ model for which most of the variation of a trait is due to a large number of QTL each with a relatively small effect.

Analyses of real data are available from dairy cattle and show zero to relatively small increases in prediction accuracy from HD genotypes. Solberg et al. [10] reported between 0.0 and 9.0% improvement in prediction accuracy across seven production and functional traits in Norwegian Red bulls. VanRaden et al. [7] found up to 6.5% (on average 0.4%) extra accuracy across 28 production traits using HD genotypes in Holstein dairy cattle.

Initially, the first factor that was suggested to affect the accuracy of genomic prediction was the LD between genome-wide SNPs [1, 2]. However, it was later shown that genomic prediction accuracy depends both on co-segregation of SNP alleles in related individuals and information from SNP alleles being in LD with QTL alleles e.g. [11]. Prediction accuracy based on LD is more persistent over distant relationships and the expectation is that higher density SNP arrays are better at capturing effects of QTL that are in LD with SNPs. Therefore, the advantage of using HD genotypes is expected to be greater for animals that are less genetically related to the reference set, and this could apply to both within-breed and across-breed genomic prediction. Thus, denser SNP genotypes may have a favorable effect on the accuracy of genomic prediction in multi-breed and crossbred populations, which are common in the sheep and beef cattle industries. Harris et al. [12] and Erbe et al. [13] showed that there was very limited improvement from using HD genotypes in across-breed prediction in Holstein and Jersey dairy cattle, but differences may be larger in sheep where breeds are genetically more related to each other and have a larger effective population size.

The objective of this study was to compare the accuracy of genomic prediction for weight, ultra-sound scanned fat and muscle traits, and wool quality and quantity traits in Australian sheep breeds based on both observed and imputed HD genotypes (600k Illumina Ovine SNP) to accuracies based on moderate-density SNP genotypes (Illumina ovine SNP50k). Using a reference set comprised of purebred, crossbred or mixed crossbred and purebred animals, prediction accuracies were compared for purebred industry sires for which very accurate estimated breeding values based on a progeny test were available. Furthermore, we contrasted accuracy of genomic prediction within a breed between animals with low and high genetic relatedness to the reference set as well as prediction within and across breeds.

## Methods

### Reference set, phenotypes and validation population

The genomic prediction reference set consisted of about 20,000 animals that were recorded for a large number of production traits measured in the “Sheep Cooperative Research Centre Information Nucleus Flock” (INF) and “Sheep Genomics Flock” (SGF). The INF consisted of eight flocks that are located across different regions of Australia and are linked to each other because artificial insemination with common sires was used between 2007 and 2011 [14]. The SGF was a single research flock located in southern New South Wales, Australia, for which data were collected between 2005 and 2006 [15]. All animals in the reference set were from multiple breeds or crossbreds with the sires comprising approximately 40% animals from Terminal breeds [Poll Dorset (PD) and White Suffolk (WS)], 20% from a Maternal breed [Border Leicester (BL)] and 40% from Merino and the dams comprising 80% Merino and 20% BL × Merino crossbreds. The dominant purebred animals were Merinos which included three sheep strains that have different wool qualities, i.e. strong wool, fine wool and ultra-fine wool types. The traits analyzed were live body weights from birth to adult age, ultra-sound scanned muscle and fat depth measured at post-weaning age and wool quantity and quality measured at yearling and adult age. The data used in this study was collected according to the guidelines of the “University of New England Animal Ethics committee” reference number AEC 09/115. The number of records and basic statistics per trait are summarized in Table 1.

**Table 1:** Summary statistics of weight, ultra sound scanned and wool traits using a multi-breed reference set

Trait	Size	Mean	SD	Range
B-WT	10,524	4.82	1.06	1.6–8.2
W-WT	12,415	27.20	7.24	7.8–43.5
PW-WT	10,881	41.52	8.79	17–75.8
Y-WT	6846	44.10	10.11	20.5–84.0
H-WT	4701	51.91	11.31	22.2–97.6
A-WT	4272	59.70	13.45	27.2–107.5
P-EMD	10,568	27.75	5.15	9.0–45.0
P-CF	9924	2.86	1.21	0.5–8.1
Y-EMD	3845	23.31	5.00	10.0–43.0
Y-CF	3841	3.12	1.31	0.6–8.5
Y-GFW	4662	3.64	1.04	1.2–7.8
Y-CFW	4423	2.46	0.65	0.93–4.76
Y-FD	3969	19.93	5.39	12.8–41.5
Y-FDCV	3554	19.26	2.86	11.7–30.8
Y-SS	3554	33.80	9.82	13.0–88.0
Y-SL	3554	80.93	13.06	38–136
A-GFW	4541	5.75	1.97	1.50–13.60
A-CFW	4540	4.19	1.39	1.13–9.91
A-FD	3001	18.17	1.84	13.80–24.60
A-FDCV	2436	18.07	2.56	11.80–27.70
A-SS	2414	36.61	10.31	3.00–68.00
A-SL	2413	98.57	18.34	41.00–149.00

*B*-*WT* birth weight, *W*-*WT* weaning weight, *PW*-*WT* post-weaning weight, *Y*-*WT* yearling weight, *H*-*WT* hogget weight, *A*-*WT* adult weight, *P*-*EMD* post-weaning eye muscle depth, *P*-*CF* post-weaning fat, *Y*-*EMD* yearling eye muscle depth, *Y*-*CF* yearling fat, *Y*-*GFW* yearling greasy fleece weight, *Y*-*CFW* yearling clean fleece weight, *Y*-*FD* yearling fibre diameter, *Y*-*FDCV* yearling fibre diameter coefficient of variation, *Y*-*SS* yearling staple strength, *Y*-*SL* yearling staple length, *A*-*GFW* adult greasy fleece weight, *A*-*CFW* adult clean fleece weight, *A*-*FD* adult fibre diameter, *A*-*FDCV* adult fibre diameter coefficient of variation, *A*-*SS* adult staple strength, *A*-*SL* adult staple length, *SD* standard deviation

A validation population was used to find the empirical accuracy of genomic prediction. The validation population was a group of industry purebred sires with accurate estimated breeding values (EBV) (accuracy ranging from 0.70 to 0.99 and on average 0.92), which were calculated based on progeny records. The phenotypes of INF and SGF animals (genomic prediction reference set) were not used in the calculation of EBV of the validation sires.

### Genotypes

The reference and validation populations were genotyped using a 50k SNP panel (Illumina Inc., San Diego, CA, USA). This 50k SNP panel provided 48,559 SNP genotypes after applying quality control based on the following criteria: individual SNP genotypes were removed if their call rates were lower than 90%, or if the GenCal (GC) scores were <0.6, if the heterozygosity rate for a given SNP deviated more than 3 SD from the population mean, if the SNP minor allele frequency was lower than 0.01, and for SNPs located on chromosomes X and Y or SNPs that deviated from Hardy–Weinberg equilibrium (P < 1 × 10^−15^). Furthermore, an individual sample was removed if the correlation of its genotypes (coded 0, 1 or 2 per locus) with those of another sample was equal or greater than 0.98.

Most of the sires and 1735 progeny from the four main breeds including Merino, BL, PD and WS were genotyped using the HD (Illumina Inc., San Diego, CA, USA) ovine SNP panel. This SNP panel provided 510,174 SNPs after applying the same quality controls as above. Using all HD genotyped animals as imputation reference set, the un-typed genotypes of the rest of the population were imputed to HD genotypes using the software program FImpute [16]. The accuracy of imputation, which was tested within subsets of animals with observed HD genotypes, was high (on average 0.98).

### Statistical methods

For the analysis based on pedigree relationships, the following mixed model was fitted using ASReml 3.0 [17]:

$${\mathbf{y}} = {\mathbf{Xb}} + {\mathbf{Z}}_{{\bf 1}} {\mathbf{a}} + {\mathbf{Ww}} + {\mathbf{Z}}_{{\bf 1}} {\mathbf{Qq}} + {\mathbf{Z}}_{{\bf 2}} {\mathbf{s}} + {\mathbf{e}},$$

where **y** is a vector of phenotypes, **b** is a vector of fixed effects, **a** is a vector of random additive polygenic effects, **w** is a vector of random maternal effects, **q** is a vector of random breed effects, **s** is a vector with random sire by flock interaction effects and **e** is a vector of random residual effects. **X**, ${\mathbf{Z}}_{{\bf 1}}$ and **W** and ${\mathbf{Z}}_{{\bf 2}}$ are incidence matrices relating fixed effect, additive genetic, maternal effects and sire by flock interaction effects to phenotypes. **Q** is a matrix with breed proportions for each animal derived from pedigree data. Up to 28 breed effects, including those of the three Merino strains, were estimated via the **Q** matrix, however the major breeds were Merino, BL, PD and WS. All random effects are identically and independently distributed except for *a* which is distributed as: $a \sim N\left( {0,{\mathbf{A}} \sigma_{a}^{2} } \right),$ where **A** is a numerator relationship and $\sigma_{a}^{2}$ is the additive genetic variance. The fixed effects in the model were birth type, rearing type, gender, age at measurement, weight at measurement and contemporary group which was defined as a cohort of site × birth year × management group. The model used for the estimation of variance components and prediction of genomic breeding values (GBV) was the same except that **A** was replaced by **G**, where **G** is a genomic relationship matrix calculated based on 50k or HD SNP genotypes using VanRaden’s [18] equation as below:

$${\mathbf{G}} = {\mathbf{MM}}^{{\prime }} / 2\sum \left( {p_{j} } \right)\left( {1 - p_{j} } \right),$$

where **M** is a matrix of the size n × m (i.e. number of individual by number of SNPs) with coefficients equal to $(2 - 2p_{j} ),\,(1 - 2p_{j} ) \,{\text{and}}\;( - 2p_{j} )$ for genotype (*A*_1_ *A*_1_), (*A*_1_ *A*_2_) and (*A*_2_ *A*_2_) of the *j*th SNP genotype respectively, $p_{j}$ is the frequency of allele *A*_1_ for the *j*th SNP genotype. $\sigma_{g}^{2}$ is the additive genetic variance estimated from SNPs. Variance components were estimated according to the restricted maximum likelihood (REML) method using either pedigree information or genomic information from 50k or HD genotypes. Genomic EBV (GEBV) were also calculated based on a Bayesian method (BayesR [13]) in which BESSiE [19] was used for prediction of GBV based on the following model:

$${\mathbf{y}} = {\mathbf{Xb}} + {\mathbf{M}}_{{\bf 1}} {\mathbf{m}} + {\mathbf{Ww}} + {\mathbf{Z}}_{{\bf 1}} {\mathbf{Qq}} + {\mathbf{Z}}_{{\bf 2}} {\mathbf{s}} + {\mathbf{e}},$$

where **m** refers to the random effects of SNPs, ${\mathbf{M}}_{{\bf 1}}$ is an incidence matrix relating SNP effects to phenotypes and the other terms are the same as described above. A mixture of four normal distributions for SNP effects with variances ${\upsigma }_{1}^{ 2} = 0,\;{\upsigma }_{2}^{ 2} = 0.0001{\upsigma }_{\text{g}}^{2} ,\;{\upsigma }_{3}^{ 2} = 0.001{\upsigma }_{\text{g}}^{2} ,$ and ${\upsigma }_{4}^{2} = 0.01{\upsigma }_{\text{g}}^{2}$ was considered in BayesR where ${\upsigma }_{\text{g}}^{2}$ is the assumed total genetic variance. The starting values for ${\upsigma }_{\text{g}}^{2}$ were taken from GREML analysis and the prior distribution of the proportion of SNPs in each distribution was the Dirichlet distribution. A total of 50,000 iterations (with 10,000 burn-in) were run for analysis.

The accuracy of GBV was assessed in a separate population of purebred industry rams including Merino, Maternal and Terminal sires (validation set), as the Pearson correlation coefficient between GBV and an accurate EBV estimated from progeny test. Correlations were estimated for each breed separately, while an effect due to the Merino strain was fitted to avoid GBV accuracy to be biased upward for merinos by evaluating accuracy across strains. The size of the validation set for different traits was 341 to 389 sires for Merino, 79 to 88 for BL, 161 to 188 for PD and 189 to 204 for WS. We also contrasted the accuracy of GBV for animals with high or low genomic relationships with the reference set. Animals with high genomic relatedness were those for which the average value of their 30 highest genomic relationships to the reference population was at least 0.20. Animals with low genetic relatedness were those for which the genomic relationship with any of the individuals in the reference set was not higher than 0.10.

## Results

### Variance components

Table 2 shows the genetic and residual variance components of the studied traits as well as the estimated heritability based on the genetic covariance matrix among animals that was estimated from pedigree or marker genotypes (50k or HD). Additive genetic variances and heritability estimates based on 50k SNP genotypes tended to be lower than those based on pedigree data (heritability was on average 4.9% lower across different traits). Other variance components including the maternal effect and the sire by site (genotype by environment) interaction effects varied little between different models and are not reported in Table 2. In most cases, estimated residual variances were slightly larger from a model based on 50k genotypes compared with those based on pedigree relationships.

**Table 2:** Additive (V_A_) and residual (V_R_) variance components and heritability estimate based on pedigree (PBLUP) and 50k (GBLUP-50k) or HD SNP genotypes (GBLUP-HD)

Trait	PBLUP			GBLUP-50k			GBLUP-HD		
	V_A_	V_R_	*h* ^2^	V_A_	V_R_	*h* ^2^	V_A_	V_R_	*h* ^2a^
B-WT	0.24	0.26	0.31	0.21	0.27	0.28	0.25	0.24	0.33
W-WT	4.62	6.62	0.36	4.13	8.36	0.27	4.77	7.95	0.31
PW-WT	8.36	15.59	0.28	7.82	15.85	0.27	9.10	15.14	0.31
H-WT	19.63	14.22	0.51	17.69	17.65	0.41	20.78	16.19	0.47
Y-WT	14.54	12.55	0.44	12.12	14.48	0.33	13.69	12.22	0.40
A-WT	27.22	26.84	0.42	26.53	28.13	0.41	30.0	26.41	0.46
P-EMD	1.32	3.73	0.26	1.41	3.68	0.26	1.56	3.57	0.28
P-CF	0.09	0.32	0.13	0.09	0.32	0.16	0.09	0.32	0.18
Y-EMD	1.56	3.49	0.31	1.97	3.15	0.39	2.04	2.89	0.41
Y-CF	0.16	0.54	0.20	0.18	0.40	0.23	0.21	0.37	0.28
Y-GFW	0.12	0.10	0.49	0.09	0.12	0.35	0.09	0.11	0.39
Y-CFW	0.06	0.06	0.45	0.07	0.08	0.42	0.07	0.07	0.46
Y-FD	1.41	0.40	0.76	1.21	0.34	0.75	1.36	0.29	0.8
Y-FDCV	3.34	2.35	0.54	2.82	2.73	0.45	3.15	2.59	0.49
Y-SL	70.7	33.28	0.67	58.51	44.98	0.56	62.02	42.09	0.59
Y-SS	29.09	50.78	0.33	19.28	55.4	0.22	22.05	54.51	0.26
A-GFW	0.34	0.26	0.55	0.32	0.33	0.47	0.34	0.3	0.51
A-CFW	0.22	0.14	0.57	0.20	0.17	0.52	0.21	0.16	0.54
A-FD	1.60	0.04	0.88	1.34	0.30	0.73	1.80	0.17	0.85
A-FDCV	2.70	2.35	0.54	2.78	2.73	0.45	2.94	2.59	0.49
A-SL	56.53	49.41	0.51	55.86	51.16	0.49	56.52	53.12	0.50
A-SS	29.62	68.34	0.28	27.68	73.79	0.26	32.19	70.53	0.30

^a^Standard error of heritability was between 0.02 and 0.09; for trait abbreviations see Table 1

Variance components estimated by using HD genotypes resulted in larger additive genetic variance, smaller residual variance and hence higher heritability across all studied traits, when compared to 50k genotypes. However, the increase in additive variance and heritability was small (up to 4% of the absolute value for heritability). Variance components and heritability estimates were similar between models that used HD genotypes and pedigree. Less than 1% differences were found between heritability estimates based on HD genotypes and pedigree when averaged across all weight, carcass scan and wool traits.

### Genomic prediction

#### Genomic prediction for weight and scanned carcass traits using a multi-breed/crossbred reference set

Tables 3, 4 and 5 show the accuracy of genomic prediction for weight and scanned carcass traits for Merino, BL, PD and WS sires, based on GBLUP (both for 50k and HD SNP genotypes) and BayesR and using the complete multi-breed reference set. Compared to 50k SNP genotypes, the HD SNP genotypes provided higher prediction accuracy but the extra accuracy was on average small. The maximum improvement in prediction accuracy as absolute value was 7.7% and was on average equal to 1.6, 1.2, 4.3 and 3.1% for Merino, BL, PD and WS sires, respectively. Terminal breeds showed a higher increase in prediction accuracy (3.7%) compared to Merino and Maternal breeds (1.4%), which suggests a tendency for greater improvement in accuracy from HD genotypes for breeds with a lower overall accuracy.

**Table 3:** Accuracy of genomic prediction of weight and scanned traits for Merino, Border Leicester (BL), Poll Dorset (PD) and White Suffolk (WS) sires based on the multi-breed reference set and GBLUP based on 50k genotypes

Trait	Size	GBLUP-50k			
		Merino	BL	PD	WS
B-WT	10,524	0.42 (0.04)^a^	0.37 (0.10)	0.10 (0.07)	0.14 (0.07)
W-WT	12,415	0.38 (0.04)	0.30 (0.10)	0.05 (0.07)	0.25 (0.07)
PW-WT	10,881	0.63 (0.04)	0.37 (0.10)	0.10 (0.07)	0.15 (0.07)
H-WT	6846	0.65 (0.04)	0.21 (0.10)	0.02 (0.07)	0.20 (0.07)
Y-WT	4701	0.61 (0.04)	0.33 (0.10)	0.20 (0.07)	0.19 (0.07)
A-WT	4272	0.66 (0.04)	0.43 (0.10)	0.00 (0.07)	0.16 (0.07)
P-EMD	10,568	0.30 (0.04)	0.23 (0.11)	0.45 (0.06)	0.35 (0.06)
P-CF	9924	0.33 (0.05)	0.31 (0.10)	0.32 (0.07)	0.27 (0.07)
Y-EMD	3845	0.39 (0.04)	0.14 (0.11)	0.14 (0.07)	0.39 (0.06)
Y-CF	3841	0.40 (0.04)	0.18 (0.11)	0.24 (0.07)	0.15 (0.07)

^a^Standard error (SE) calculated according to $\left( {\frac{{1 - r^{2} }}{n - 2}} \right)^{0.5}$ where *r* is the correlation coefficient and *n* is the number of paired observations; for trait abbreviations see Table 1

**Table 4:** Accuracy of genomic prediction of weight and scanned traits for Merino, Border Leicester (BL), Poll Dorset (PD) and White Suffolk (WS) sires based on the multi-breed reference set and GBLUP based on HD genotypes

Trait	Size	GBLUP-HD			
		Merino	BL	PD	WS
B-WT	10,524	0.43 (0.04)^a^	0.41 (0.10)	0.12 (0.07)	0.17 (0.07)
W-WT	12,415	0.38 (0.04)	0.29 (0.11)	0.13 (0.07)	0.33 (0.06)
PW-WT	10,881	0.64 (0.04)	0.37 (0.10)	0.15 (0.07)	0.20 (0.07)
H-WT	6846	0.67 (0.04)	0.21 (0.11)	0.04 (0.07)	0.23 (0.07)
Y-WT	4701	0.63 (0.04)	0.33 (0.10)	0.25 (0.07)	0.20 (0.07)
A-WT	4272	0.68 (0.04)	0.42 (0.10)	0.06 (0.07)	0.21 (0.07)
P-EMD	10,568	0.31 (0.05)	0.19 (0.11)	0.50 (0.06)	0.40 (0.06)
P-CF	9924	0.33 (0.05)	0.31 (0.10)	0.37 (0.07)	0.29 (0.07)
Y-EMD	3845	0.43 (0.04)	0.21 (0.11)	0.14 (0.07)	0.39 (0.06)
Y-CF	3841	0.42 (0.04)	0.22 (0.11)	0.24 (0.07)	0.15 (0.07)

^a^Standard error (SE) calculated according to: $\left( {\frac{{1 - r^{2} }}{n - 2}} \right)^{0.5}$ where *r* is the correlation coefficient and *n* is the number of paired observations; for trait abbreviations see Table 1

**Table 5:** Accuracy of genomic prediction of weight and scanned traits for Merino, Border Leicester (BL), Poll Dorset (PD) and White Suffolk (WS) sires based on the multi-breed reference set and BayesR based on HD genotypes

Trait	Size	BayesR			
		Merino	BL	PD	WS
B-WT	10,524	0.43 (0.04)^a^	0.40 (0.10)	0.11 (0.07)	0.17 (0.07)
W-WT	12,415	0.38 (0.04)	0.28 (0.11)	0.13 (0.07)	0.34 (0.07)
PW-WT	10,881	0.64 (0.04)	0.35 (0.10)	0.21 (0.07)	0.22 (0.07)
H-WT	6846	0.65 (0.04)	0.22 (0.11)	0.06 (0.07)	0.23 (0.07)
Y-WT	4701	0.66 (0.04)	0.27 (0.11)	0.27 (0.07)	0.29 (0.07)
A-WT	4272	0.68 (0.04)	0.42 (0.10)	0.11 (0.07)	0.21 (0.07)
P-EMD	10,568	0.31 (0.04)	0.21 (0.11)	0.49 (0.06)	0.40 (0.06)
P-CF	9924	0.32 (0.04)	0.30 (0.10)	0.40 (0.07)	0.27 (0.07)
Y-EMD	3845	0.43 (0.04)	0.18 (0.11)	0.15 (0.07)	0.40 (0.06)
Y-CF	3841	0.42 (0.04)	0.20 (0.11)	0.24 (0.07)	0.15 (0.07)

^a^Standard error (SE) calculated according to: $\left( {\frac{{1 - r^{2} }}{n - 2}} \right)^{0.5}$ where *r* is the correlation coefficient and *n* is the number of paired observations; for trait abbreviations see Table 1

When using HD genotypes, the accuracy of genomic prediction was very similar between GBLUP and BayesR across all traits, with an average absolute value of the difference in genomic prediction accuracy between GBLUP-HD and BayesR of −0.008, −0.006 and 0.03 for Merino, Maternal and Terminal breeds, respectively.

#### Genomic prediction for wool traits in Merino based on a Merino reference set

Table 6 shows the accuracy of genomic prediction of breeding value for wool traits in Merino sires based on GBLUP—with 50k and HD SNP density, and BayesR using HD SNP density with only Merinos in the reference set. The extra accuracy resulting from HD genotypes ranged from 0.0 to 8.0% with an average of 5.0%. No considerable difference in accuracy was observed between GBLUP and BayesR.

**Table 6:** Accuracy of genomic prediction of wool traits in Merino sheep based on GBLUP (50k/HD) and BayesR

Trait	Size	GBLUP-50k	GBLUP-HD	BayesR
Y-GFW	4662	0.68 (0.03)^a^	0.69 (0.03)	0.67 (0.03)
Y-CFW	4423	0.62 (0.03)	0.63 (0.03)	0.63 (0.03)
Y-FD	3969	0.69 (0.03)	0.75 (0.03)	0.72 (0.03)
Y-FDCV	3554	0.46 (0.04)	0.47 (0.04)	0.47 (0.04)
Y-SL	3554	0.56 (0.03)	0.62 (0.03)	0.63 (0.03)
Y-SS	3554	0.33 (0.04)	0.41 (0.04)	0.43 (0.04)
A-GFW	4541	0.65 (0.03)	0.69 (0.03)	0.69 (0.03)
A-CFW	4540	0.59 (0.03)	0.63 (0.03)	0.62 (0.03)
A-FD	3001	0.61 (0.03)	0.67 (0.03)	0.74 (0.03)
A-FDCV	2436	0.32 (0.04)	0.36 (0.04)	0.36 (0.04)
A-SL	2414	0.59 (0.04)	0.67 (0.04)	0.66 (0.04)
A-SS	2413	0.40 (0.04)	0.46 (0.04)	0.45 (0.04)

^a^Standard Error (SE) calculated according to: $\left( {\frac{{1 - r^{2} }}{n - 2}} \right)^{0.5}$ where *r* is the correlation coefficient and *n* is the number of paired observations; for trait abbreviations see Table 1

#### Genomic prediction within and across breeds from purebred or crossbred reference sets

Table 7 shows the accuracy of genomic prediction within and across breeds for three weight traits and two scanned carcass traits. Using HD genotypes and a purebred Merino reference set resulted in a small increase in GBV accuracy (0.0 to 2.5%) for Merino sires, which was similar to the increase in genomic prediction accuracy in Tables 3, 4 and 5. A larger increase (0.3 to 9.6%) was observed for Merino sires based on prediction from crossbred Merinos. However, it should be noted that the magnitude of the prediction accuracy for Merino sires from crossbred Merinos is still much lower than the prediction from purebred Merinos.

**Table 7:** Accuracy of genomic prediction within and across breeds from purebred or crossbred reference set

Trait	Reference set	Size	GBV accuracy (50k)				GBV accuracy (HD)			
			Merino	BL	PD	WS	Merino	BL	PD	WS
B-WT	Mer	3159	0.42 (0.04)^a^	−0.14 (0.11)	−0.16 (0.07)	−0.015 (0.07)	0.43 (0.04)	0.08 (0.11)	−0.09 (0.07)	0.09 (0.07)
	BL × Mer	1187	0.37 (0.04)	0.25 (0.11)	0.06 (0.07)	0.073 (0.07)	0.38 (0.04)	0.28 (0.11)	0.06 (0.07)	0.11 (0.07)
	PD × Mer (A)	1616	0.35 (0.04)	−0.09 (0.11)	0.25 (0.07)	0.056 (0.07)	0.38 (0.04)	0.01 (0.11)	0.25 (0.07)	0.06 (0.07)
	WS × Mer (B)	1015	0.33 (0.04)	−0.04 (0.11)	0.04 (0.07)	0.152 (0.07)	0.34 (0.04)	0.01 (0.11)	0.04 (0.07)	0.20 (0.07)
	(A) + (B)	2631	0.39 (0.04)	−0.02 (0.11)	0.25 (0.07)	0.163 (0.07)	0.40 (0.04)	−0.01 (0.11)	0.26 (0.07)	0.18 (0.07)
W-WT	Mer	4586	0.36 (0.04)	−0.09 (0.11)	−0.01 (0.07)	0.001 (0.07)	0.37 (0.04)	0.10 (0.11)	0.02 (0.07)	0.01 (0.07)
	BL × Mer	1495	0.31 (0.04)	0.33 (0.10)	−0.10 (0.07)	−0.047 (0.07)	0.34 (0.04)	0.33 (0.11)	−0.09 (0.07)	0.06 (0.07)
	PD × Mer (A)	936	0.24 (0.05)	0.10 (0.11)	0.10 (0.07)	0.045 (0.07)	0.34 (0.04)	0.10 (0.11)	0.13 (0.07)	0.14 (0.07)
	WS × Mer (B)	876	0.23 (0.05)	0.03 (0.11)	−0.08 (0.07)	0.218 (0.07)	0.32 (0.04)	0.04 (0.11)	0.00 (0.07)	0.33 (0.07)
	(A) + (B)	1812	0.32 (0.04)	0.02 (0.11)	0.19 (0.07)	0.117 (0.07)	0.41 (0.04)	0.02 (0.11)	0.27 (0.07)	0.19 (0.07)
PW-WT	Mer	3935	0.50 (0.04)	−0.01 (0.11)	−0.03 (0.07)	0.076 (0.07)	0.52 (0.04)	−0.01 (0.11)	0.01 (0.07)	0.10 (0.07)
	BL × Mer	1824	0.40 (0.04)	0.36 (0.10)	−0.02 (0.07)	−0.026 (0.07)	0.41 (0.04)	0.37 (0.11)	0.09 (0.07)	0.13 (0.07)
	PD × Mer (A)	1849	0.39 (0.04)	0.01 (0.11)	0.28 (0.07)	0.021 (0.07)	0.46 (0.04)	0.00 (0.11)	0.31 (0.07)	0.07 (0.07)
	WS × Mer (B)	1224	0.33 (0.04)	0.00 (0.11)	0.02 (0.07)	0.230 (0.07)	0.35 (0.04)	0.06 (0.11)	0.08 (0.07)	0.28 (0.07)
	(A) + (B)	3073	0.47 (0.04)	−0.01 (0.11)	0.27 (0.07)	0.251 (0.07)	0.54 (0.04)	−0.01 (0.11)	0.31 (0.07)	0.28 (0.07)
P-EMD	Mer	3449	0.337 (0.04)	−0.059 (0.11)	0.084 (0.07)	0.074 (0.07)	0.337 (0.04)	−0.062 (0.11)	0.084 (0.07)	0.101 (0.07)
	BL × Mer	1602	0.241 (0.04)	0.217 (0.11)	0.124 (0.07)	0.028 (0.07)	0.244 (0.04)	0.232 (0.11)	0.144 (0.07)	0.102 (0.07)
	PD × Mer (A)	1809	0.270 (0.04)	0.004 (0.11)	0.150 (0.07)	0.037 (0.07)	0.284 (0.04)	0.002 (0.11)	0.174 (0.07)	0.042 (0.07)
	WS × Mer (B)	1249	0.190 (0.04)	0.000 (0.11)	0.044 (0.07)	0.134 (0.07)	0.201 (0.04)	0.000 (0.11)	0.046 (0.07)	0.141 (0.07)
	(A) + (B)	2544	0.250 (0.04)	0.001 (0.11)	0.160 (0.07)	0.152 (0.07)	0.254 (0.04)	0.002 (0.11)	0.181 (0.07)	0.157 (0.07)
PW-CF	Mer	2685	0.314 (0.04)	0.076 (0.11)	0.073 (0.07)	−0.099 (0.07)	0.318 (0.04)	0.091 (0.11)	0.024 (0.07)	−0.005 (0.07)
	BL × Mer	1186	0.136 (0.05)	0.240 (0.11)	0.044 (0.07)	0.044 (0.07)	0.139 (0.04)	0.253 (0.11)	0.064 (0.07)	0.065 (0.07)
	PD × Mer (A)	1295	0.134 (0.05)	0.121 (0.11)	0.296 (0.07)	0.069 (0.07)	0.138 (0.04)	0.126 (0.11)	0.322 (0.07)	0.080 (0.07)
	WS × Mer (B)	1250	0.130 (0.05)	0.000 (0.11)	0.001 (0.07)	0.074 (0.07)	0.133 (0.04)	0.000 (0.11)	0.003 (0.07)	0.116 (0.07)
	(A) + (B)	2540	0.170 (0.05)	0.021 (0.11)	0.286 (0.07)	0.076 (0.07)	0.184 (0.04)	0.024 (0.11)	0.296 (0.07)	0.121 (0.07)

*BL* Border Leicester, *PD* Poll Dorset, *WS* White Suffolk

^a^Standard error (SE) calculated according to: $\left( {\frac{{1 - r^{2} }}{n - 2}} \right)^{0.5}$ where *r* is the correlation coefficient and *n* is the number of paired observations; for trait abbreviations see Table 1

The data in Table 7 can be used to infer the accuracy of genomic prediction across breeds. The increase in genomic prediction accuracy for BL, PD or WS sires from a purebred Merino reference set, which is genetically distant to the target breeds, was low and showed a small non-significant improvement in prediction accuracy when moving from 50k to HD prediction. However, genomic prediction of PD and WS sires based on a combined crossbred reference set (PD × M + WS × M) showed a greater improvement in prediction accuracy (up to 8.0%). It should be noted that this accuracy was still low, even when using HD genotypes.

#### Genomic prediction for animals highly or lowly related to the reference set

**Fig. 1:**
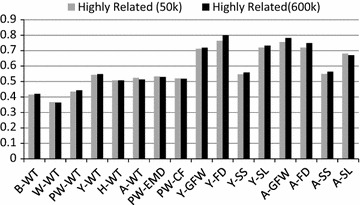
Accuracy of genomic prediction for animals that are genetically highly related to the reference set based on GBLUP using 50k or HD marker genotypes

**Fig. 2:**
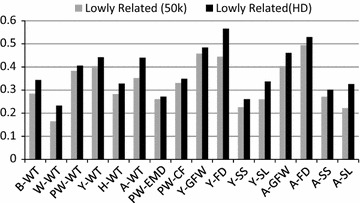
Accuracy of genomic prediction for animals that are genetically lowly related to the reference set based on GBLUP using 50k or HD marker genotypes

Figures 1 and 2 compare the accuracy of genomic prediction for two groups of Merino sires used as validation animals, one with a high and one with a low genomic relationship to the purebred Merino reference set. For highly related animals, the gain in accuracy from using HD genotypes was very low (on average 0.8%) but it was significantly higher for lowly related animals (up to 12% and on average 5.2%).

#### Regression of EBV on GBV

Table 8 shows the regression coefficient of the accurate (>0.90) breeding values that were based on progeny data on the estimated genomic breeding values. Regression coefficients estimates were between 0.74 and 0.94 and were on average higher for GBLUP or BayesR methods based on HD SNPs compared to GBLUP based on moderate density SNPs. No significant difference in regression coefficient was observed between GBLUP and BayesR prediction methods based on HD SNPs.

**Table 8:** Regression coefficient of genomic breeding values from accurate (>90%) pedigree breeding values for wool traits based on GBLUP 50k and HD and BayesR

Trait	GBLUP-50k	GBLUP-HD	BayesR
Y-GFW	0.85	0.83	0.83
Y-CFW	0.82	0.88	0.87
Y-FD	0.81	0.86	0.86
Y-FDCV	0.74	0.77	0.76
Y-SL	0.81	0.88	0.88
Y-SS	0.75	0.77	0.78
A-GFW	0.86	0.94	0.93
A-CFW	0.82	0.85	0.85
A-FD	0.79	0.80	0.80
A-FDCV	0.83	0.83	0.84
A-SL	0.85	0.87	0.86
A-SS	0.78	0.79	0.80

For trait abbreviations see Table 1

## Discussion

This study investigates the possible improvement in accuracy of genomic prediction of breeding values for weight, scanned carcass and wool quantity and quality traits in Australian sheep when using high-density SNP genotypes. First, we compared the variance components that were estimated based on relationships derived from 50k and HD genotypes to those based on pedigree relationships. Estimated additive genetic variances based on HD genotypes were larger than those based on the 50k SNP panel, which suggests that the HD panel captures more genetic variation; this is likely due to higher LD between SNPs and QTL. Estimated genetic variances based on the HD panel were similar while the estimates based on the 50k panel were lower than those based on pedigree data. However, the **A** and **G** matrices are not necessarily on the same scale (e.g. the **G** matrix is derived as a genomic relationship) so these estimates cannot be directly compared. Haile-Mariam et al. [20] also reported that the additive genetic variances and heritabilities estimated from Bovine50k genotypes were lower than those based on pedigree BLUP for 29 production traits in Australian dairy cattle. Legarra [25] argued that the relationship matrices used to estimate genetic variances should be comparable, i.e. the same average relationship and the same average inbreeding. In any case, the difference between 50k and HD panels is the most relevant comparison and this is not affected by scaling.

HD SNP panels provided higher prediction accuracies but the increase had only practical significance for individuals that were not closely related to the reference population. The average improvement in prediction accuracy was small, ~2.2% which is likely due to the effect of closer relationships providing information that is not much improved by higher marker density. SNPs can capture co-segregation of alleles (family relationships) as well as the LD between SNPs and QTL [5, 11, 21, 22]. Co-segregation is based on linkage between SNPs and QTL which exists over much larger chromosomal regions, therefore not requiring a very high SNP density for adequate prediction. Van der Werf et al. [22] pointed out that prediction from closer relatives is similar to prediction in populations with a lower effective size in which fewer effective chromosome segments are segregating. This observation leads to the same conclusion, i.e. that higher SNP density will have a limited effect on the prediction accuracy when the relationship between reference and target set is stronger.

Previous reports based on real data in dairy cattle also showed a very limited improvement in prediction accuracy when using HD genotypes [7, 8], which confirm results from some simulation studies [12, 23]. However, Meuwissen and Goddard [6] showed a larger gain in prediction accuracy, using a simulation model that included more QTL with large effects, e.g. all the genetic variation of a polygenic trait was due to three to 30 QTL segregating on one chromosome. Meuwissen and Goddard [6] and Clark et al. [9] showed that the use of denser SNP panels was more beneficial if traits are controlled by fewer QTL with larger effects. Our results show limited extra accuracy from HD genotypes, which could indicate that the distribution of QTL effects is closer to the infinitesimal model assumption.

Genomic prediction in a multi-breed reference set could potentially benefit from across-breed prediction when using HD genotypes, as has been suggested in various studies [24, 26, 27]. However, we observed only a small (from 0 to a slightly positive value) increase in accuracy when using information from other breeds. Across-breed prediction could be lower due to differences in both QTL and SNP allele frequencies, incomplete LD between SNPs and QTL across breeds and different allele substitution effects at QTL in different breeds, e.g. due to epistatic interactions [28]. Using higher density SNPs would address only the incomplete LD aspect but not the other two factors. In this study, a slightly greater improvement in GBV accuracy from using HD genotypes was observed for purebred Merinos (5%) based on a Merino reference set compared to a larger multi-breed reference set. Very limited prediction accuracy from HD genotypes was found for PD and WS breeds based on the Merino sheep reference set, which is likely due to the large genetic distance between Merino and PD or WS as terminal breeds. These results are in line with those of other across-breed prediction studies, e.g. [12, 27] who reported small to no across-breed prediction accuracy from a combined Holstein and Jersey dairy cattle reference set. Interestingly, our results showed a notable (on average 5%) improvement in genomic prediction of PD or WS sheep based on a combined crossbred PD or WS reference set. This suggests that HD SNP panels could be useful to improve LD between SNPs and QTL within diverse breeds or between closely-related breeds, in which case it is also more likely that QTL effects are similar. However, predictions across more distant breeds will not benefit from HD genotypes due to lower levels of LD and possibly larger differences in QTL effects.

Some studies have shown that using moderate-density SNP panels (~50k) provide a more marked improvement in genomic prediction accuracy over low-density SNP panels in different livestock species. Moghaddar et al. [29] compared prediction based on panels of 5k, 10k, 20k and 50k SNPs and showed on average a 11 to 13% gain in prediction accuracy for different production traits in Merino sheep. In dairy cattle, Moser et al. [30] reported on average 10% extra accuracy by switching from very low-density SNP genotypes (3000 to 5000) to moderate-density SNP genotypes (50k). Other studies have also reported relatively large improvements in prediction accuracy from using moderate-density SNP panels compared to low-density SNP sets [3, 5, 31]. However, this study showed improvements in prediction accuracy from using ovine HD genotypes compared to moderate-density genotypes (ovine 50k) seems generally much smaller, but significant improvements were still observed for individuals distantly related to the reference population. This is consistent with the theory about genomic prediction accuracy [32].

The regression coefficient of EBV on GBV was on average higher (less biased) based on HD SNPs than on 50k SNPs. This could be related to the larger additive genetic variances that were estimated when using HD genotypes and are more similar to the estimates of additive genetic variance based on pedigree data. Bias could also occur if selected SNPs were used for genomic prediction. To some extent, the BayesR method uses selected SNPs, in the sense that it uses some priors to emphasize a larger effect for some SNPs by giving them more weight. However, regression coefficients did not differ between GBV based on GBLUP using HD genotypes and GBV based on the BayesR method, which suggests that this explanation is less likely.

Regression coefficients of EBV on GBV were generally lower than 1.00 (0.74 to 0.94). This may be due to the **G**-matrix not being expressed at the same scale as the numeric relationship matrix (**A**) used in the genetic evaluation that produces the EBV, or because of differences in the method for accounting for genetic groups in the reference and validation populations. The **A**-matrix is based on pedigree relationships whereas GBV are calculated with a **G**-matrix that uses relationships across various subpopulations within the population. Since this study was mainly aimed at evaluating genomic prediction accuracy, we did not attempt to rescale the **G**-matrix, since accuracy is calculated as a correlation which is independent of scale. Furthermore, the averages of diagonal and off-diagonal elements of **A** and **G** were similar (1.01 and 0.00 for **A**, 1.00 and 0.00 for **G** based on 50k SNP density and 1.03 and 0.00 for **G** based on HD density) as was suggested by Legarra [25] as a requirement to obtain unbiased estimation of breeding values.

## Conclusions

Our results show that the use of high-density (600k) SNP genotypes for the genomic prediction of weight and wool production traits in a multi-breed sheep population resulted in a small improvement in accuracy compared to a moderate SNP density (50k). Improvement in accuracy was greater for individuals that were distantly related to the reference set. Prediction accuracy based on a reference set from other breeds was low and showed limited improvement with HD genotypes. Results of GBLUP and BayesR were not significantly different.
